# Biomechanical Changes in the Lower Limb After a Quadriceps Fatigue Task in Association With Dynamic Knee Valgus

**DOI:** 10.7759/cureus.87390

**Published:** 2025-07-06

**Authors:** Makoto Asaeda, Yukio Mikami, Akihiro Matsumoto, Takeya Araki, Kiyo Ueda, Noriaki Maeda, Tomoya Onishi, Hideyuki Ito, So Miyahara, Atsuo Nakamae, Nobuo Adachi

**Affiliations:** 1 Department of Rehabilitation Medicine, Hiroshima University Hospital, Hiroshima, JPN; 2 Faculty of Wakayama Health Care Sciences, Takarazuka University of Medical and Health Care, Wakayama, JPN; 3 Department of Sports Rehabilitation, Graduate School of Biomedical and Health Sciences, Hiroshima University, Hiroshima, JPN; 4 Department of Orthopedic Surgery, Graduate School of Biomedical and Health Sciences, Hiroshima University, Hiroshima, JPN

**Keywords:** anterior cruciate ligament, electromyography, fatigue, knee valgus, lower limb biomechanics

## Abstract

Objectives: Dynamic knee valgus (DKV), characterized by knee abduction and external rotation, has been associated with anterior cruciate ligament (ACL) injury, but the predictive accuracy of DKV for ACL injury remains unclear.

Methods: This controlled laboratory study examined fatigue effects on lower limb biomechanics and electromyography (EMG) during jump landing in healthy men. Participants were categorized into induced (n=11) and non-induced (n=8) DKV groups based on changes in knee abduction moment after a fatigue task.

Results: Compared to the non-induced DKV group, the induced group exhibited a significantly reduced hip flexion angle (p=0.030), lower vertical ground reaction force (p=0.040), and decreased quadriceps peak EMG amplitude (p=0.008), along with a significant increase in hip internal rotation moment (p=0.001). Receiver operating characteristic analysis revealed that hip internal rotation moment had the highest discriminative power for classifying DKV occurrence, with an area under the curve (AUC) of 0.994 (95% CI: 0.960-1.000), followed by hip adduction moment (AUC = 0.896) and quadriceps peak amplitude (AUC = 0.883).

Conclusions: Findings suggest that DKV occurrence after fatigue is associated with altered quadriceps activity and hip rotation mechanics. These insights may inform ACL injury prevention strategies.

## Introduction

Dynamic knee valgus (DKV), characterized by knee abduction and external rotation [1], has been identified as a potential predictor of anterior cruciate ligament (ACL) injury. Prospective studies have reported that higher knee abduction moments during jump-landing tasks are associated with an increased risk of ACL injury [2]. However, systematic reviews and meta-analyses have shown inconsistent results regarding the predictive value of DKV for ACL injury [3].

Fatigue is a well-recognized risk factor for ACL injuries because it can compromise neuromuscular control and alter lower limb biomechanics [4]. For example, fatigue can affect joint moments at the hip and knee and modify ground reaction forces (GRF) during landing tasks [5-9]. These changes may increase the likelihood of adopting a DKV posture, yet the extent to which fatigue contributes to DKV occurrence remains unclear. Moreover, it is unknown whether fatigue-related DKV depends on individual baseline neuromuscular characteristics.

The hip muscles, particularly the gluteus medius (GM), play a critical role in maintaining hip stability and controlling knee alignment during landing. Weakness or fatigue of the gluteal muscles can reduce hip control, potentially increasing DKV risk [10]. Although quadriceps function is also important for landing stability, the interplay between hip and knee musculature during fatigue-induced DKV is not fully understood. Previous studies have examined electromyography (EMG) activity of the gluteal and quadriceps muscles under fatigue conditions [11], but the findings have been inconsistent. Some studies report that fatigue of the gluteal muscles can lead to increased knee valgus moments during landing [7,12], while others find minimal effects on quadriceps and hamstring EMG activity [13]. This inconsistency highlights the need for further research to clarify the role of lower limb muscle activation in DKV following fatigue [6].

To address these gaps, this study aimed to investigate the effects of a knee flexion-extension fatigue task on lower limb biomechanics and EMG during jump landing in healthy male participants. Participants were classified into induced and non-induced DKV groups based on changes in knee abduction moment after fatigue. We hypothesized that participants with induced DKV exhibit distinct alterations in lower limb biomechanics and muscle activity patterns compared to those without induced DKV.

## Materials and methods

Methods

Study Design

This controlled laboratory study was approved by the Institutional Research Ethics Committee of Takarazuka University of Medical and Health Care (approval number: 2009251). The study was conducted in accordance with the Declaration of Helsinki. All participants provided written informed consent prior to participation.

Participants

Healthy male participants aged 18 to 22 years who engaged in competitive-level sports activities (Tegner activity scale >7 [14]) at least three times per week were recruited via posters. The Tegner Activity Scale was used to assess participants' activity level. This scale is freely available for research use and does not require permission or licensing for academic publication purposes. Eligible participants were required to be able to understand the study instructions and to have no history of current lower limb joint disease or neurological disorders. Individuals were excluded if they had a body mass index (BMI) of 30 kg/m² or higher, experienced pain or limited range of motion in the lower limb joints, had a history of musculoskeletal injuries or abnormalities in the lower limbs, or participated in sports that involved repetitive high-impact landings, such as basketball or volleyball. A priori sample size estimation was not conducted; however, a post hoc power analysis was performed based on the observed effect size (Cohen’s d = 0.65) for between-group differences in hip internal rotation moment, yielding a statistical power of 0.80 at an alpha level of 0.05.

Study Protocol

All testing was performed in a controlled laboratory environment. Participants underwent a standardized warm-up prior to testing.

Single-Leg Drop Landing (Pre-fatigue)

Participants wore tight-fitting clothing to which 39 infrared reflective markers (diameter: 10 mm) were attached in accordance with the Plug-in Gait model of the Vicon Motion System. The jump landing motion consisted of a single-leg drop landing from a height of 20 cm. Before the fatigue task, participants practiced this movement three times to familiarize themselves with the task. For the test, they performed three successful landings while maintaining a stable landing posture for 5 s each. During these trials, marker position data and GRFs were collected using a three-dimensional motion analysis device (Vicon MX, Vicon Motion Systems, UK) and two force plates (AMTI, Massachusetts, USA). The camera frequency was set to 100 Hz, and the GRF was recorded at 1000 Hz. Marker data were processed using a 6-Hz low-pass Butterworth fourth-order filter.

Fatigue Task

Immediately following the pre-fatigue single-leg drop landings, participants performed the fatigue task without rest. The fatigue protocol involved isokinetic knee extension/flexion movements using Biodex Medical Systems (New York, USA). The task was performed in a seated position with the chair tilted at 8°, and the participant held the chair’s bars with both upper limbs. The attachment was placed distal to the left lower leg (2 cm proximal to the medial malleolus), and the axis of rotation was aligned with the femoral condyles. Gravity correction was performed at 20° knee flexion. Participants were seated deeply with approximately one finger’s width of space between the seat and the back of the lower leg. After this positioning, they were securely fixed using waist, shoulder, and thigh belts. Participants then performed maximum effort knee extensions from 135° flexion to 0° extension, followed by knee flexions, at an angular velocity of 120°/s for 40 repetitions each. This protocol was based on previous studies that confirmed its high reliability [15]. Objective fatigue was assessed by calculating the percentage decrease in peak torque from the first peak (repetition 1-3) to the last peak (repetition 37-40). Subjective fatigue was evaluated using the Borg scale. The use of the Borg CR scale® is based on Borg's original work [16] and is reproduced with permission from BorgPerception AB (License Agreement #11HBOCH#). The fatigue task protocol used in this study was originally developed by the authors and does not require permission for use or reproduction.

Single-Leg Drop Landing (Post-fatigue)

Immediately after completing the fatigue task, participants performed the single-leg drop landing protocol again without any additional practice trials. The same procedures for marker placement, motion capture, and GRF collection were used as in the pre-fatigue trials.

Data Analysis

A single trained examiner performed all measurements; however, data analysis was performed by an independent investigator blinded to group assignment.

Lower limb kinematics and kinetics during single-leg drop landing were analyzed using the Plug-in Gait model (Vicon Motion System). Marker position data and GRFs were collected during each trial. The initial contact (IC) was defined when the vertical GRF exceeded 10 N. Kinematic and kinetic data, including the lateral, anteroposterior, and vertical components of the GRF at maximum vertical GRF, were extracted for analysis. For each participant, three successful trials were averaged.

Changes in lower limb kinematics and kinetics after the fatigue task were calculated by subtracting pre-fatigue values from post-fatigue values. The knee adduction/abduction moment was analyzed as a key component of DKV, and the change in this moment was used to classify participants: those with a positive change in DKV were defined as the induced DKV group, and those with a negative change were defined as the non-induced DKV group. Comparisons of lower limb biomechanics and EMG were then made between the two groups.

EMG Analysis

For EMG measurements, we used the Trigno Avanti Sensor Wireless System (Delsys Inc., USA), with the sensors attached to the left quadriceps and GM, and set at a sampling frequency of 2000 Hz and bandwidth of 20-450 Hz, according to a previous study [17]. IC was defined as the point at which the vertical acceleration was indicated immediately before the point at which the built-in acceleration sensor indicated the maximum downward acceleration [18]. The analysis interval was set to 100 ms after IC. In the EMG in the analysis interval, an offset from the signal, root mean square (window length: 0.1, window laps: 0.09), and maximum voluntary isometric contraction (performed in the lateral recumbent position with 10° hip abduction for 5 s) were removed. The maximum value at 100 ms from the IC was calculated as the peak EMG amplitude. Power spectrum analysis (window length: 0.1, window laps: 0.09) was performed from row EMG data. The average value of three jump landings (average median power frequency (MDF)) was calculated, and the difference between the peak EMG amplitude and mean MDF before and after the fatigue task during jump landing was calculated.

Statistical Analysis

IBM SPSS Statistics for Windows, Version 27 (Released 2020; IBM Corp., Armonk, New York, United States) was used for statistical analysis. An unpaired t-test was used to compare induced and non-induced DKV when normality and homoscedasticity were observed; otherwise, the Wilcoxon rank-sum test was used. For items that showed a significant difference between the two groups, receiver operating characteristic (ROC) analysis was performed to calculate the area under the curve (AUC), with parameters showing significant differences between the two groups as the explanatory factors and whether DKV was induced or not as the target factor. The AUC was 0.7, indicating “acceptable or better” [19]. The significance level was defined at <5%. A post hoc power analysis was conducted based on the observed effect size (Cohen’s d = 0.65) for the between-group difference using G*Power (version X.X, Universität Düsseldorf, Germany)

## Results

Participants and degree of fatigue

Eleven participants were assigned to the induced DKV group and eight to the non-induced DKV group. There were no significant differences in demographic characteristics such as age, height, weight, or BMI between the groups (Table 1), indicating baseline comparability. Similarly, no significant group differences were found in quadriceps peak torque or subjective fatigue ratings during the fatigue task, suggesting that the fatigue protocol was consistently applied. Despite experiencing comparable levels of fatigue, DKV developed only in the induced group, suggesting that factors beyond the magnitude of fatigue may have contributed to the emergence of DKV.

**Table 1:** Participant characteristics. Statistical analysis: independent (unpaired) t-test. BMI, body mass index; DKV, dynamic knee valgus; SD, standard deviation

	Induced DKV (n=11)	Non-induced DKV (n=8)	Test Statistic	p-value
	Mean ± SD	Mean ± SD		
Age (years)	20.09 ± 0.83	19.57 ± 0.98	t=-1.210	0.244
Height (cm)	173.15 ± 6.98	172.06 ± 6.95	t=-0.323	0.751
Weight (kg)	66.55 ± 10.51	61.00 ± 8.23	t=-1.180	0.255
BMI	22.10 ± 2.23	20.54 ± 1.58	t=-1.610	0.127
First peak (Nm/kg)	2.21 ± 0.26	1.99 ± 0.31	t=-1.598	0.130
Last peak (Nm/kg)	1.00 ± 0.31	0.96 ± 0.17	t=-0.356	0.727
Borg scale (point)	17.27 ± 1.79	16.00 ± 2.24	t=-1.335	0.200

Lower-limb kinematics and kinetics

A comparative analysis of joint angles and moments revealed distinct movement pattern alterations between groups. Notably, the hip flexion angle showed a divergent trend: participants in the non-induced DKV group exhibited increased flexion following fatigue, while those in the induced group showed decreased flexion (Table 2).

**Table 2:** Changes in lower-limb kinematics during landing before and after the fatigue protocol. Data are presented as mean ± standard deviation or median (25th–75th percentile), as appropriate based on data distribution. p-values were calculated using the independent-samples t-test for normally distributed data and the Wilcoxon rank-sum test for non-normally distributed data. ^a^ Wilcoxon rank-sum test; DKV, dynamic knee valgus; IQR, interquartile range; SD, standard deviation The items listed in the table show positive values.

		Induced DKV (n=11)		Non-induced DKV (n=7)		Test Statistic	p-value	Effect size
		Mean ± SD	Median (IQR)	Mean ± SD	Median (IQR)			
Ankle (degree)	Dorsi flexion	-0.88 ± 3.27	-2.09 (-3.10 to 1.71)	0.01 ± 1.72	0.01 (-2.09 to 1.87)	t=0.661	0.518	0.320
	Inversion	-0.15 ± 0.46	-0.35 (-0.46 to 0.15)	0.57 ± 1.02	0.19 (-0.03 to 1.77)	U=30	0.441^a^	0.181
	Internal rotation	0.67 ± 2.67	1.78 (-0.66 to 2.40)	-4.18 ± 6.68	-1.03 (-13.51 to 0.16)	U=27	0.298^a^	0.246
Knee (degree)	Flexion	2.74 ± 4.15	3.33 (-1.62 to 6.49)	1.72 ± 1.85	-0.06 (-0.24 to 0.07)	t=-0.607	0.552	0.294
	Adduction	0.00 ± 2.77	-0.75 (-2.65 to 2.49)	-0.03 ± 2.45	0.16 (0.09 to 0.26)	t=-0.023	0.982	0.011
	Internal rotation	0.39 ± 2.12	0.13 (-0.82 to 2.18)	6.01 ± 8.20	0.03 (-0.01 to 0.09)	U=36	0.821^a^	0.053
Hip (degree)	Flexion	3.17 ± 4.29	2.25 (-0.26 to 4.98)	-1.38 ± 3.29	-1.53 (-3.67 to 2.03)	t=-2.539	0.030	1.155
	Adduction	-0.22 ± 2.52	0.06 (-2.72 to 1.98)	0.47 ± 3.84	0.85 (-1.63 to 3.86)	t=0.464	0.649	0.224
	Internal rotation	-0.29 ± 3.65	-0.71 (-2.81 to 1.11)	-1.14 ± 3.12	-1.83 (-2.29 to 0.81)	t=-0.510	0.617	0.247

The induced DKV group also demonstrated reduced hip adduction moment and vertical GRF, whereas the non-induced group exhibited increases in these parameters. This pattern suggests a reduction in frontal plane loading and potentially altered force attenuation strategies among those who developed DKV.

Additionally, the hip rotation moment shifted toward internal rotation in the induced group and toward external rotation in the non-induced group, indicating altered transverse plane control in response to fatigue (Table 3). The increase in hip internal rotation moment observed in the induced DKV group is consistent with previous studies reporting increased hip internal rotation angles following fatigue tasks [20-23].

**Table 3:** Changes in lower-limb kinetics during landing before and after the fatigue protocol. Data are presented as mean ± standard deviation or median (25th–75th percentile), as appropriate based on data distribution. p-values were calculated using the independent-samples t-test for normally distributed data and the Wilcoxon rank-sum test for non-normally distributed data. ^a^ Wilcoxon rank-sum test; DKV, dynamic knee valgus; GRF, ground reaction force; IQR, interquartile range; SD, standard deviation The items listed in the table show positive values.

		Induced DKV (n=11)		Non-induced DKV (n=7)		Test Statistic	p-value	Effect size
		Mean ± SD	Median (IQR)	Mean ± SD	Median (IQR)			
Ankle (Nm/kg)	Dorsi flexion	0.13 ± 0.32	0.05 (-0.05 to 0.42)	0.06 ± 0.03	0.06 (0.03 to 0.08)	U=19	0.077^a^	0.416
	Inversion	0.01 ± 0.05	0.00 (-0.03 to 0.07)	0.01 ± 0.14	0.03 (-0.11 to 0.13)	U=35	0.751^a^	0.075
	Internal rotation	-0.02 ± 0.08	-0.01 (-0.08 to 0.01)	0.05 ± 0.03	0.05 (0.01 to 0.08)	t=2.059	0.056	0.995
Knee (Nm/kg)	Flexion	-0.20 ± 0.44	-0.06 (-0.34 to 0.16)	-0.05 ± 0.28	-0.06 (-0.24 to 0.07)	t=0.792	0.440	0.383
	Adduction (DKV)	-0.19 ± 0.14	-0.14 (-0.26 to -0.06)	0.23 ± 0.20	0.16 (0.09 to 0.26)	t=5.083	< 0.001	2.458
	Internal rotation	0.00 ± 0.08	-0.02 (-0.04 to 0.02)	0.03 ± 0.07	0.03 (-0.01 to 0.09)	t=0.770	0.452	0.372
Hip (Nm/kg)	Flexion	0.28 ± 0.20	0.25 (0.09 to 0.48)	0.10 ± 0.20	0.15 (0.08 to 0.20)	t=-1.855	0.082	0.897
	Adduction	-0.26 ± 0.18	-0.25 (-0.35 to -0.05)	0.05 ± 0.19	-0.03 (-0.10 to 0.25)	t=3.420	0.004	1.653
	Internal rotation	0.03 ± 0.07	0.01 (-0.02 to 0.09)	-0.09 ± 0.04	-0.08 (-0.10 to -0.07)	t=-4.053	0.001	1.960
GRF (N/kg)	Frontal	0.04 ± 0.36	0.34 (-5.85 to 6.46)	0.22 ± 0.27	2.13 (-0.28 to 9.27)	t=1.099	0.288	0.531
	Sagittal	-0.03 ± 0.47	0.41 (-1.78 to 5.71)	0.02 ± 0.43	3.19 (-0.56 to 11.68)	t=0.236	0.817	0.114
	Vertical	-1.01 ± 1.51	-8.00 (-17.06 to 1.99)	0.49 ± 1.15	2.81 (-5.06 to 14.27)	t=2.234	0.040	0.108

The observed reduction in hip flexion angle in the DKV group may reflect a compensatory strategy to reduce eccentric quadriceps loading, potentially minimizing joint strain during landing. Previous studies have reported inconsistent findings regarding hip flexion post-fatigue, with some indicating increases [24,25] and other decreases [20]. Such inconsistencies may stem from differences in participant characteristics, task design, or fatigue protocols.

Regarding the hip adduction moment, the observed decrease in the induced DKV group may indicate reduced external hip adduction force, possibly influenced by changes in vertical GRF or compensatory hip mechanics. Although GM EMG activity did not differ significantly, subtle neuromuscular adaptations not captured by surface EMG may underlie this change. Interestingly, no significant differences in ankle and knee joint kinematics or kinetics were observed between the groups. This could be attributed to methodological factors such as marker placement accuracy or signal filtering settings. Future studies should explore these aspects in greater detail to clarify their potential influence.

EMG analysis

EMG analysis revealed a significantly lower quadriceps peak amplitude after the fatigue task in the induced DKV group (Table 4). This diminished activation may reflect impaired neuromuscular response capacity, potentially contributing to insufficient stabilization during landing. In contrast, no significant differences were observed in GM activity between the groups.

**Table 4:** EMG of the quadriceps and gluteus medius during landing. Data are presented as mean ± standard deviation or median (25th–75th percentile), depending on the data distribution. p-values were calculated using the independent-samples t-test for normally distributed data and the Wilcoxon rank-sum test (a) for non-normally distributed data. Comparisons were made between the DKV-induced and non-DKV groups. ^a^ Wilcoxon rank-sum test; DKV, dynamic knee valgus; IQR, interquartile range; MVC, maximum voluntary contraction; SD, standard deviation

			Induced DKV (n=11)		Non-induced DKV (n=7)		Test Statistic	p-value	Effect size
			Mean ± SD	Median (IQR)	Mean ± SD	Median (IQR)			
Quadriceps	Peak amplitude (%MVC)	Before	42.63 ± 14.29	36.47 (32.63 to 55.43)	47.06 ± 18.05	50.99 (29.07 to 58.18)	U=28	0.570^a^	0.280
		After	52.89 ± 28.45	39.99 (34.97 to 58.33)	65.35 ± 25.07	65.63 (39.75 to 80.57)	t=0.946	0.008	0.630
		Change	10.26 ± 24.17	5.05 (-3.63 to 23.34)	18.29 ± 29.67	2.40 (-7.87 to 49.07)	t=0.629	0.538	0.304
	Median frequency (Hz)	Before	63.45 ± 15.08	63.75 (46.28 to 77.43)	74.06 ± 8.10	74.14 (67.50 to 80.50)	t=1.700	0.108	0.822
		After	54.94 ± 14.90	61.51 (42.05 to 65.13)	60.01 ± 16.95	57.93 (43.36 to 74.23)	t=0.666	0.515	0.322
		Change	-8.51 ± 7.34	-8.03 (-15.70 to -1.23)	-14.05 ± 19.64	-10.97 (-27.53 to -4.93)	t=-0.859	0.403	0.415
Gluteus medius	Peak amplitude (%MVC)	Before	58.77 ± 27.62	56.82 (41.69 to 74.82)	72.20 ± 24.79	71.58 (55.00 to 87.50)	U=24	0.312^a^	0.505
		After	61.69 ± 45.24	45.85 (34.01 to 75.7)	81.84 ± 41.88	68.55 (49.07 to 123.33)	U=29	0.390^a^	0.203
		Change	2.92 ± 46.36	4.87 (-22.81 to 10.95)	9.64 ± 19.23	0.53 (-4.00 to 35.83)	U=35	0.751^a^	0.075
	Median frequency (Hz)	Before	54.45 ± 13.07	51.88 (47.85 to 54.01)	67.93 ± 13.82	67.37 (60.32 to 81.68)	U=33	0.618^a^	0.117
		After	54.90 ± 8.26	53.48 (48.77 to 57.01)	73.44 ± 19.84	63.43 (58.26 to 89.81)	t=2.780	0.441^a^	0.181
		Change	0.45 ± 16.98	3.73 (-4.83 to 8.68)	5.51 ± 13.92	0.67 (-5.47 to 18.87)	t=0.658	0.497	0.160

ROC analysis

**Figure 1:**
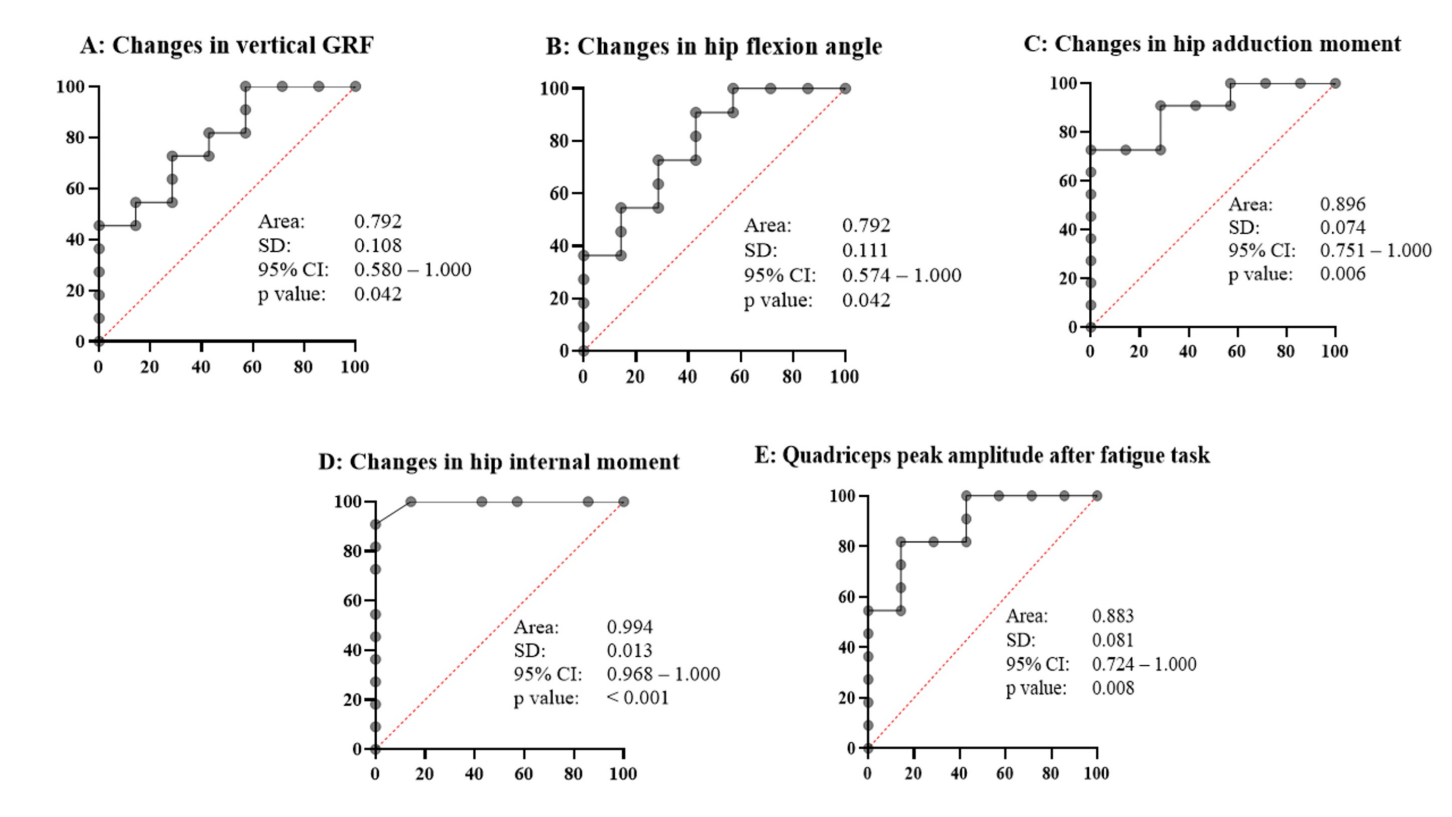
Receiver operating characteristic analysis with the parameters showing significant differences between the two groups. A: Changes in vertical GRF, B: Changes in hip flexion angle, C; Changes in hip adduction moment, D: Changes in hip internal moment, E: Quadriceps peak amplitude after fatigue task AUC, area under the curve; CI, confidence interval; GRF, ground reaction force; SD, standard deviation

ROC analysis was conducted to evaluate the discriminatory capacity of key biomechanical and neuromuscular variables. Hip internal rotation moment demonstrated the highest classification performance (AUC = 0.994). Hip adduction moment and quadriceps peak amplitude also exhibited excellent discriminatory ability (AUC = 0.896 and 0.883, respectively). Both vertical GRF and hip flexion angle showed good classification performance (AUC = 0.792 for both, Figure 1). However, the exceptionally high AUC for the hip internal rotation moment raises concerns about potential overfitting or collinearity. As the ROC analysis was conducted within the same sample without cross-validation, these findings should be interpreted with caution.

Overall, participants in the induced DKV group exhibited a combination of reduced hip flexion angle, decreased quadriceps peak amplitude, and significantly lower hip adduction moment. These findings suggest distinct alterations in lower limb biomechanics and neuromuscular activation under fatigued conditions.

## Discussion

This study investigated the effects of fatigue on lower limb biomechanics and muscle activation patterns during jump landing in healthy men. Despite comparable levels of subjective and objective fatigue between groups, the findings suggest that the development of DKV under fatigue may be influenced by factors beyond fatigue intensity alone, such as individual neuromuscular control or sensorimotor regulation.

Consistency with previous findings

The findings of this study are consistent with several previous observations. Notably, the decrease in hip flexion angle following fatigue aligns with trends commonly reported in the literature. According to a systematic review by Jayalath et al. [26], jump landing after general fatigue is often characterized by increased knee flexion and decreased hip flexion. Although this study did not observe significant changes in knee angles, a clear reduction in hip flexion was noted.

Furthermore, movement patterns such as knee valgus and excessive femoral internal rotation are widely recognized as typical fatigue-induced adaptations [27]. Thomas et al. [9] reported that after inducing quadriceps and hamstring fatigue, participants landed with significantly greater hip internal rotation at initial contact and less knee flexion, indicative of a stiffer landing. These findings mirror the increased hip internal rotation moment and reduced hip flexion angle found in the DKV group of this study.

Reduced contribution from the hip abductors also plays a critical role. Prior research on female athletes has shown that decreased hip abduction strength is associated with increased knee valgus displacement during landing [28], supporting the present study’s finding that reduced hip adduction moment is linked to DKV.

Recent work by Hodel et al. [29] further supports this mechanism, demonstrating that individuals with greater hip internal rotation range of motion and weaker external rotators show increased knee valgus displacement during fatigued landings. This reinforces the idea that hip external rotator and abductor dysfunction may underlie the emergence of DKV.

Points of divergence from previous studies

Some differences from prior studies were also observed. For instance, several investigations have reported increased knee and hip flexion during fatigue as an adaptive strategy to absorb impact, particularly in handball players [26]. While this may appear contradictory, the shared observation of reduced hip flexion in both contexts suggests a shift toward a knee-dominant landing strategy. This indicates that multiple compensatory patterns may exist under fatigue, varying by individual and task.

Unlike many previous studies that analyzed all participants as a single group and reported average trends [30], this study categorized participants into induced and non-induced DKV groups. While some studies observed increased muscle activation under fatigue as a compensatory mechanism, the present study found reduced activation in a specific subgroup-the DKV group. This suggests that individuals predisposed to DKV may demonstrate unique neuromuscular and biomechanical responses to fatigue. In contrast, the non-DKV group appeared able to maintain effective hip control under fatigue.

Perspectives on fatigue-induced DKV and preventive strategies

Knee valgus has often been explained in terms of local joint alignment or ligament laxity. However, when fatigue is taken into account, a more comprehensive mechanism emerges, i.e., compromised neuromuscular control at proximal joints (e.g., the hip) may reduce distal joint stability (e.g., the knee), contributing to the onset of DKV.

Fatigue may limit the ability to adequately flex the hip and reduce stabilization by the pelvic musculature, including the hip abductors and external rotators. In response, the body may compensate with knee valgus, femoral internal rotation, or excessive tibial external rotation to maintain postural balance. Although inefficient, these compensations may act as a last-resort strategy when muscular support diminishes, shifting loading to passive structures and increasing stress in unfavorable directions, such as valgus and rotational forces, thereby elevating ACL injury risk.

This study’s focus on hip-knee coordination under fatigue, rather than on the knee in isolation, is a significant contribution. DKV may reveal latent motor control deficits, including fatigue intolerance in the hip external rotators or limited quadriceps endurance, that do not appear under non-fatigued conditions.

From a preventive standpoint, rehabilitation and training strategies should not only target static alignment correction or isolated muscle strengthening. Rather, they should emphasize maintaining hip and knee control during fatigue. Such interventions could include endurance training for hip abductors and external rotators, fatigue resistance training for the quadriceps, and plyometric training to optimize landing mechanics.

Limitations

This study has several limitations. First, it included only male participants, limiting the generalizability of the findings. Second, the small sample size may have reduced statistical power and external validity. Third, the lack of synchronization between the EMG and motion capture systems restricted the analysis of muscle activation timing. Fourth, the focus on only the quadriceps and GM excluded assessment of other relevant muscle groups [31]. Additionally, sex differences in landing biomechanics and fatigue responses, such as higher knee adduction moments observed in men [32] and sex-specific fatigue adaptations [33], were not addressed. Finally, variability in sport type and competitive level among participants may have influenced landing mechanics [34].

## Conclusions

This study demonstrated that individuals who developed DKV following a fatigue-inducing task exhibited specific alterations in lower limb biomechanics and muscle activation patterns, including reduced hip flexion, decreased hip adduction and internal rotation moments, lowered vertical ground reaction force, and diminished quadriceps activity. These findings suggest that DKV development may be linked to fatigue-related impairments in proximal joint control and neuromuscular function. The ability of these variables to distinguish between those who developed DKV and those who did not highlights their potential as early indicators for identifying individuals at risk. These insights may contribute to the development of targeted interventions aimed at enhancing hip control and quadriceps function to mitigate DKV-related injury risks in physically active populations.
